# New species and new records in Cerambycidae (Coleoptera) of the state of Bahia, Brazil

**DOI:** 10.3897/zookeys.517.10219

**Published:** 2015-08-12

**Authors:** Maria Helena M. Galileo, Ubirajara R. Martins, Antonio Santos-Silva

**Affiliations:** 1 PPG Biologia Animal, Departamento de Zoologia, Universidade Federal do Rio Grande do Sul, Porto Alegre, RS, Brazil; 2 Museu de Zoologia, Universidade de São Paulo, Caixa Postal 42594, 04218-970, São Paulo, São Paulo, Brazil; 3 Fellow of the Conselho Nacional de Desenvolvimento Científico e Tecnológico

**Keywords:** Neotropical, new records, new species, taxonomy

## Abstract

Two new species are described from Bahia (Brazil): *Coleoxestia beckeri* (Cerambycini), and *Oncioderes obliqua* (Onciderini). Nine species are recorded for the first time for Bahia (Brazil). Key to species of *Oncioderes* Martins & Galileo, 1990 is provided. *Coleoxestia beckeri* is included in a previous key.

## Introduction

The study of the specimens sent by Vitor O. Becker from the state of Bahia in Brazil allowed the description of two new species, and also new records for the state. Many specimens were caught near Boa Nova, a small city located in the south central region of Bahia, in area of Caatinga, a kind of desert vegetation, common in northeastern Brazil. Previously, Martins et al. (2015) in a work about the cerambycid fauna of Bahia state, described 12 new species and recorded 52 species for the first time to that state.

## Material and methods

Photographs were taken with a Canon EOS Rebel T3i DSLR camera, Canon MP-E 65mm f/2.8 1-5X macro lens, controlled by Zerene Stacker AutoMontage software. Measurements were taken in ‘‘mm’’ using a micrometer ocular Hensoldt/Wetzlar - Mess 10 in the Leica MZ6 stereomicroscope, also used in the study of the specimen.

The identification of specimens was carried out with the aid of reference collection of the MZSP, with comparison between types, photographs of types, original descriptions, and redescriptions.

The collection acronyms used in this study are as follows:

CVOB Collection Vitor O. Becker, Camacan, Bahia, Brazil;

MZSP Museu de Zoologia, Universidade de São Paulo, São Paulo, Brazil.

## Systematics

### Cerambycinae Latreille, 1802
Cerambycini Latreille, 1802
Sphallotrichina Martins & Monné, 2002

#### Coleoxestia beckeri sp. n.

Taxon classificationAnimaliaColeopteraCerambycidae

http://zoobank.org/66B24B09-CC6B-4162-B1DD-D0746E19E77D

Figs 1–7

##### Description.

**Figures 1–7.**
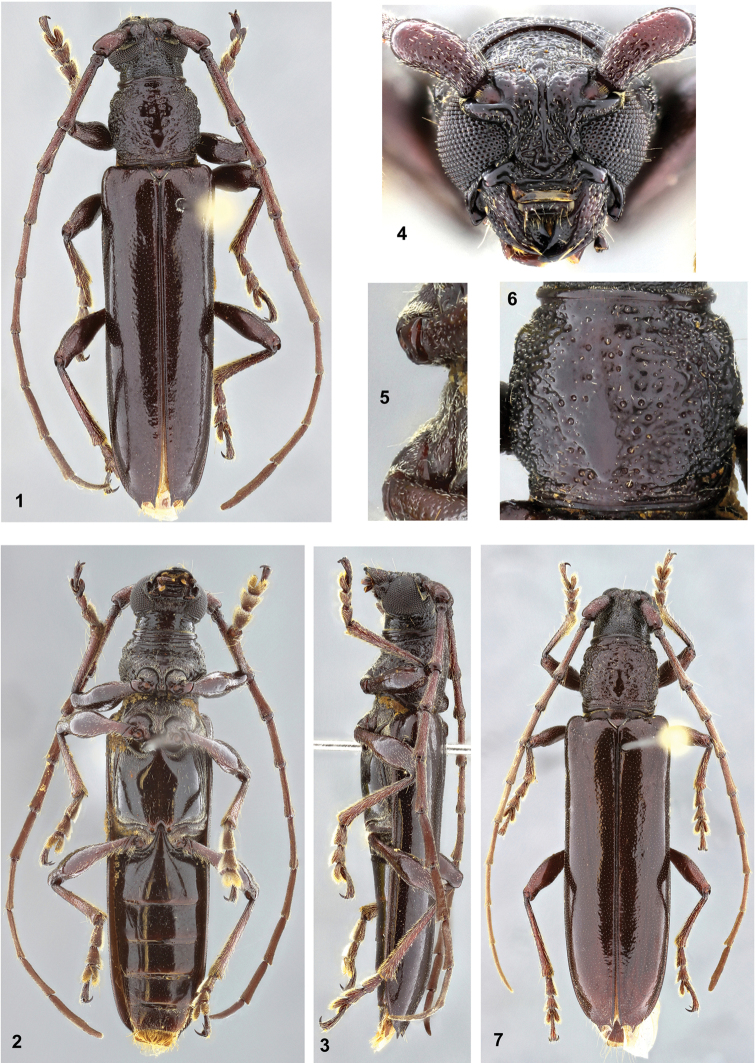
*Coleoxestia beckeri*. **1–6.** Holotype male: **1** dorsal view **2** ventral view **3** lateral view **4** head, frontal view **5** prosternal and mesosternal process, lateral view **6** pronotum **7** female paratype, dorsal view.

Holotype male (Figs 1–6). Integument dark brown, almost black on head and prothorax; elytra black on narrow band along suture and apical spines; scutellum blackish, with reddish-brown macula about center; antennomeres dark reddish-brown, except for narrow blackish area on apex of III–VI.

Head. Frons coarsely, confluently punctate, laterally with narrow carina from anterior margin to antennal tubercle (more distinct towards the latter); with very short, sparse setae. Area between antennal tubercles somewhat finely punctate; with very short, sparse setae. Area between upper eye lobes with elongate, smooth, glabrous tubercle, narrowed towards vertex; on each side of tubercle moderately coarsely, confluently punctate, with short, sparse setae. Vertex coarsely, partially confluently punctate, punctures finer towards prothoracic margin; with very short, sparse setae. Longitudinal sulcus distinct from clypeus to area between antennal tubercles, deeper towards the latter. Area behind upper eye lobes coarsely, confluently punctate; with very short and sparse setae, denser, interspersed with some long setae close to superior margin of eye. Area behind lower eye lobes longitudinally sulcate about middle; area close to eyes finely, abundantly punctate, with short setae interspersed with long setae; area close to prothorax moderately finely and sparsely punctate, almost glabrous. Antennal tubercles moderately finely punctate (frontally punctures finer, denser). Gula laterally transversely sulcate, moderately finely, sparsely punctate (center smooth). Submentum opaque, finely, abundantly punctate, interspersed with coarse punctures; anteriorly transversely sulcate close to anterior margin; anterior margin narrow, elevate; with short, abundant setae. Postclypeus moderately coarsely, abundantly punctate close to frons, laterally and anteriorly smooth; area close to frons depressed; punctate area with short setae and one long seta on each side. Labrum finely punctate; centrally with short setae, anteriorly and laterally with long setae. Mandibles laterally coarsely, moderately abundantly punctate; with short setae interspersed with some long setae. Distance between upper eye lobes equal to 0.40 times length of scape; distance between lower eye lobes, in frontal view, equal to 0.65 times length of scape. Antennae as long as 1.6 times elytral length; reaching the apex at middle of antennomere XI. Scape slightly enlarged towards apex; shiny, moderately coarsely, densely, confluently punctate on base, gradually finer, sparser towards apex (apex dorsally smooth and glabrous); with short moderately sparse setae interspersed with some long setae. Antennomeres III–IV straight, nodose at apex; outer side without carina and distinct sensorial area; finely, abundantly punctate (punctures denser, coarser laterally and ventrally), except for smooth apex; with short setae, ventrally and around apical smooth area interspersed with long setae. Antennomere V somewhat microsculptured, apex nodose, laterally slightly carinate; sensorial area slightly distinct at distal third; setae as on III and IV (short setae slightly denser). Antennomeres VI-X microsculptured, pubescent; outer apex dentate (slightly at VI); outer side carinate; sensorial area wide, from base to apex (dorsally less distinct on basal third of VI). Antennomere XI microsculptured, pubescent; not distinctly divided at area of constriction of outer side. Antennal formula (ratio) based on antennomere III: scape = 0.62; pedicel = 0.15; IV = 0.64; V = 0.73; VI = 0.76; VII = 0.76; VIII = 0.73; IX = 0.73; X = 0.71; XI = 1.00.

Thorax. Prothorax as long as 0.95 times largest width; laterally with three, not strong gibbosities. Pronotum coarsely punctate (vermiculate on some areas), except for smooth central sub-elliptical callosity, from basal fifth to about anterior third; around sub-elliptical callosity, punctures notably coarse, with fine punctures inside (mainly on anterior half); punctures denser on posterolateral sides; with very short, sparse setae, laterally denser. Lateral sides of prothorax coarsely vermiculate-punctate, except for anterior area finely punctate, interspersed with coarse punctures; with short, moderately sparse setae. Basal half of prosternum laterally coarsely, abundantly punctate, with short setae; center of basal half smooth, glabrous; anterior half coarsely, transversely striate, with short, very sparse setae. Prosternal process centrally smooth, glabrous on basal half; remaining surface with short setae; apex vertically inclined, concave in lateral view. Mesosternum and mesepisterna pubescent. Mesepimera pubescent towards elytra, distinctly less so towards procoxal cavity. Mesosternal process without tubercle, pubescent. Metepisterna pubescent. Metasternum with narrow band of pubescence close to metepisterna, meso- and metacoxal cavities; remaining surface with very short and sparse setae, interspersed with some long setae; finely, sparsely punctate. Scutellum triangular. Elytra. Shiny, finely, moderately abundantly punctate (mainly on basal half), except for narrow, slightly coarser, denser punctate area close to apex; with very short, sparse setae (invisible depending on angle of light); apex with two spines with similar size. Legs. Femora with short, sparse setae, longer, more abundant on ventral side of peduncle (mainly on metafemora); apex of femora rounded.

Abdomen. Ventrites I–IV centrally smooth, glabrous (ventrite I with long, very sparse setae; IV with very short, sparse setae and fine, sparse punctures), laterally with short setae close to margin. Ventrite V slightly shorter than IV; finely, sparsely punctate, interspersed with some coarse punctures; with short, sparse setae, laterally and posteriorly denser, somewhat longer; apex widely sub-truncate.

Female (Fig. 7). Antennae as long as 1.4 times elytral length; reaching elytral apex. Ventrites I–IV with long, sparse setae, laterally narrowly pubescent. Ventrite V slightly longer than IV; moderately finely, abundantly punctate (distinctly sparser on center of basal half); apex rounded. Mesepimera pubescent.

Variation. Integument reddish-brown (mainly elytra); scutellum without reddish-brown macula; mesepimera in male pubescent throughout; legs totally or partially reddish-brown.

##### Dimensions in mm

**(holotype/male/female).** Total length, 17.8/14.2–17.8/19.2–22.0; length of prothorax at center, 3.1/2.5–3.0/3.3–3.7; largest width of prothorax, 3.3/2.8–3.3/3.6–4.0; anterior width of prothorax, 2.5/2.1–2.6/2.7–3.0; posterior width of prothorax, 2.9/2.3–2.9/3.0-3.4; humeral width, 3.8/3.3–4.0/4.4-5.1; elytral length, 12.6/9.9–12.7/13.7–15.3.

##### Type material.

Holotype male from BRAZIL, *Bahia*: 9 km W Boa Nova (“Caatinga”, 14°36'S, 40°26'W, 750 m), 4–8.XII.2013, V. O. Becker col. (MZSP). Paratypes (all from BRAZIL, *Bahia*): same data as holotype, 8 males (CVOB); Porto Seguro (Arraial d’Ajuda; 16°27'S, 39°03'W; 40 m), 3 males (CVOB), 4 females (3 CVOB, 1 MZSP), 23.XI.2013, V. O. Becker col. (MZSP); Aracatu (“Fazenda Lagoa do Tamburi”, Caatinga, 14°30,961'S, 41°27,512'W), female, 18.X.2012, A. S. Ferreira col. (MZSP).

##### Etymology.

Named after Vitor Osmar Becker, collector of the type series.

##### Remarks.

*Coleoxestia beckeri* sp. n. is similar to *Coleoxestia nigropicea* (Bates, 1870), but differs as follows: frons coarsely, abundantly punctate; apex of scape not projected; pronotum with short setae; pronotum (Fig. 6) less coarsely punctate; elytra shiny. In *Coleoxestia nigropicea* (see figs. 309–312 by Martins and Monné 2005; or fig. 14 by Fragoso 1993) the frons is not coarsely punctate, the scape is projected at inner side of apex, the pronotum is glabrous and coarser punctate, and the elytra is opaque on base. It differs from *Coleoxestia atrata* (Gounelle, 1909) by the vertex more abundantly punctate, by the antennae in male distinctly surpassing the elytral apex, and by the pronotum not transversely sulcate and with short setae. In *Coleoxestia atrata* the vertex is sparsely punctate or almost smooth, the antennae in male not surpassing the elytral apex, and the pronotum is transversely sulcate and glabrous.

*Coleoxestia beckeri* can be included in the alternative of couplet “33” from Martins and Monné (2005), considering the elytra as “black” (translated; modified):

**Table:** 

33(32)	Pronotum almost without wrinkles (not distinctly sulcate)	**33**’
–	Pronotum with wrinkles (distinctly sulcate) or densely punctate with some wrinkles	**34**
33’(33)	Scape projected at inner side of apex. Brazil (Pará)	***Coleoxestia nigropicea* (Bates, 1870)**
–	Scape without projection at inner side of apex. Brazil (Bahia)	***Coleoxestia beckeri* sp. n.**

### LAMIINAE Latreille, 1825
Onciderini Thomson, 1860

#### Oncioderes obliqua sp. n.

Taxon classificationAnimaliaColeopteraCerambycidae

http://zoobank.org/97BA23A9-299E-42E3-9BAA-9913A4E7985B

Figs 8–11

##### Description.

Holotype female. Integument black.

Head. Frons elongate, centrally somewhat tumid; microsculptured, without punctures; pubescence centrally testaceous, short, moderately dense (distinctly sparser on area close to lateral band); lateral area close to eyes with narrow, yellowish band, from clypeus to apex of antennal tubercles. Central region between antennal tubercles with glabrous, smooth, irregular area. Postclypeus finely, abundantly punctate, except for lateral smooth area; laterally with yellowish band of pubescence, centrally with moderately wide central band of pubescence, areas between bands of pubescence with sparse yellowish setae; laterally, near anterior margin, with dark, long, sparse setae; distal margin with fringe of yellowish setae. Labrum basally with transverse band with short, testaceous pubescent, centrally with wide, transverse band with yellowish pubescence (longer than on base), distally with narrow band golden pubescence; lateral sides of area with yellowish pubescence with long, dark, sparse setae. Coronal suture distinct from clypeus to anterior margin of prothorax (less distinct from middle of eyes to prothorax). Antennal tubercles microsculptured; with short, testaceous pubescence on each side of central yellowish band (sparser on some areas). Area between upper eye lobes with short, brown, dense pubescence. Vertex and area behind eyes with dense, short, yellowish pubescence. Area behind lower eye lobes with sparse row of punctures (each puncture with dark, long, thick seta). Genae as long as 0.65 times lower eye lobes; laterally coarsely, sparsely punctate, with short, moderately abundant, yellowish setae; frontally moderately coarsely, confluently punctate, except for smooth area close to clypeus and frons, with moderately sparse, yellowish-brown setae, except for glabrous area close to clypeus and frons. Distance between upper eye lobes equal to 0.35 times length of scape. Lower eyes lobes large, oblong; distance between them, in frontal view, equal to 0.70 times length of scape. Antennae as long as 1.5 times elytral length; reaching elytral apex at apex of antennomere IX; scape gradually expanded to apex, with brown pubescence, ventrally with short, sparse setae at distal half; antennomeres with whitish-gray pubescence, gradually brownish towards distal antennomeres; antennomeres ventrally with sparse, dark setae throughout (mainly III); antennal formula (ratio) based on antennomere III: scape = 0.87; pedicel = 0.19; IV = 0.69; V = 0.52; VI = 0.48; VII = 0.46; VIII = 0.44; IX = 0.39; X = 0.31; XI = 0.33.

Thorax. Prothorax transverse; lateral sides with distinct tubercle near middle. Pronotum with two transverse, large, sub-fused gibbosities about middle of each side; center with large gibbosity, connected to the basal lateral gibbosity; pubescence yellowish-brown, distinctly not obliterating integument, interspersed with whitish pubescence on some areas. Lateral sides of prothorax with pubescence as on pronotum, but distinctly sparser. Prosternum and prosternal process with abundant, yellowish pubescence, distinctly longer than on pronotum. Mesosternum with yellowish-brown pubescence, not obliterating integument. Mesepisterna with yellowish pubescence, longer and denser than on mesosternum. Metepisterna with yellowish-brown pubescence. Metasternum with yellowish-brown pubescence, laterally and close to metacoxae more yellowish. Scutellum with yellowish-brown pubescence. Elytra. Base with transverse band with yellowish-brown pubescence; from humerus to about middle, oblique, wide band with brownish pubescence, attaining suture; laterally from humerus to about apex of basal quarter, with brownish pubescence connected with oblique band at humerus; centrally, on basal third, between oblique bands, triangular area with yellowish-white pubescence; on distal half with large, elliptical macula with black pubescence on its basal third, brownish towards apex, except for small, irregular yellowish-white macula at base of brownish pubescence; remaining surface with whitish-gray pubescence, except for narrow area on apex with brownish pubescence, interspersed with yellowish; coarsely, deeply, sparsely punctate; apices rounded together. Legs. With dense, yellowish-white pubescence.

Abdomen. Ventrites coarsely, sparsely punctate (more distinctly on I–II); with yellowish-brown pubescence; ventrite V about as long as IV, trapezoidal, with apex slightly emarginate at center.

**Figures 8–11.**
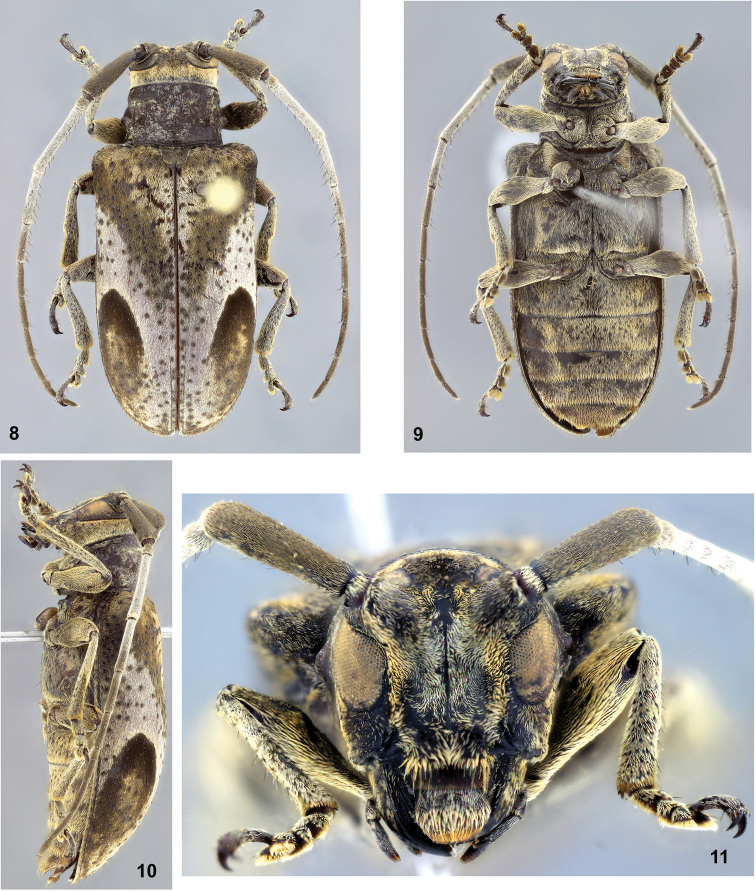
*Oncioderes obliqua*, holotype female: **8** dorsal view **9** ventral view **10** lateral view **11** head, frontal view.

##### Dimensions in mm

**(female).** Total length, 15.5; length of prothorax at center, 2.5; largest width of prothorax (between apices of tubercles), 4.2; anterior width of prothorax, 3.5; posterior width of prothorax, 3.8; humeral width, 6.5; elytral length, 11.5.

##### Type material.

Holotype female from BRAZIL, *Bahia*: 9 km W Boa Nova (“Caatinga”, 14°36'S, 40°26'W, 750 m), 4-8.XII.2013, V. O. Becker col. (MZSP).

##### Etymology.

Latin, *obliqua* = oblique (feminine adjective); allusive to the oblique band with yellowish-brown pubescence on elytra.

##### Remarks.

*Oncioderes obliqua* sp. n. is similar to *Oncioderes piauiensis* Martins & Galileo, 2013, but differs as follows: more robust body; pubescence of head, pronotum and elytra shorter and less compact (mainly on pronotum); lateral tubercle of prothorax more distinct; elytra with oblique band from humerus to about middle; elytral punctures sparser. In *Oncioderes piauiensis*, the body is slender, the pubescence of head, pronotum and elytra are longer and more compact, the lateral tubercle of prothorax is smaller, the elytra have not oblique band from humerus to middle, and the elytral punctures are more abundant.

#### Key to species of *Oncioderes*

**Table:** 

1	Lateral tubercle of prothorax very distinct; elytra with wide and oblique band with yellowish-brown pubescence from humerus to about middle. Brazil (Bahia)	***Oncioderes obliqua* sp. n.**
–	Lateral tubercle of prothorax poorly elvated; elytra without wide and oblique band	**2**
2(1)	Elytra with small, abundant, irregular white maculae of pubescence. Brazil (São Paulo)	***Oncioderes picta* Martins & Galileo, 1990**
–	Elytral pubescence distinctly more compact, forming large maculae	**3**
3(2)	Elytral pubescence predominantly orangish-yellow. Brazil (Piauí)	***Oncioderes piauiensis* Martins & Galileo, 2013**
–	Elytral pubescence predominantly yellowish-white. Brazil (Rondônia)	***Oncioderes rondoniae* Martins & Galileo, 1990**

### New records from Bahia (BRAZIL)

**CERAMBYCINAE Latreille, 1802**

**CERAMBYCINI Latreille, 1802**

**CERAMBYCINA Latreille, 1802**

***Juiaparus batus lacordairei* (Gahan, 1892)**. Material examined. BRAZIL, *Bahia*: 9 km W Boa Nova (“Caatinga”, 14°36'S, 40°26'W, 750 m), male, 4–8.XII.2013, V. O. Becker col. (CVOB).

This species was described from Argentina (Buenos Aires and Salta); currently it is recorded from Brazil (Goiás, Minas Gerais, Rio de Janeiro, São Paulo, Paraná, Santa Catarina), Bolivia, Paraguay, Argentina (Jujuy, Salta, Tucumán, Santiago del Estero, Chaco, Santa Fé, Córdoba), and Uruguay (Monné 2015a). Monné (2015a) did not record three provinces in Argentina: Buenos Aires, from where it was described the female syntype; and Misiones, recorded by Bosq (1934), Viana (1972), and Martins and Monné (2002). Viana (1972) also recorded the species from Guiana, and Mato Grosso (Brazil) (places not recorded in Monné 2015a). Martins and Monné (2002) did not exclude those places, only mentioning that Viana (1972) has listed them. Thus, formally, Guiana, the state of Mato Grosso in Brazil, and the provinces of Buenos Aires, Misiones and Corrientes in Argentina need to be added to the list by Monné (2015a).

**EBURIINI Blanchard, 1845**

***Eburodacrys lenkoi* Napp & Martins, 1980**. Material examined. BRAZIL, *Bahia*: 9 km W Boa Nova (“Caatinga”, 14°36'S, 40°26'W, 750 m), male, 4–8.XII.2013, V. O. Becker col. (CVOB).

*Eburodacrys lenkoi* was described and remains known only from Brazil (Minas Gerais, Espírito Santo, Rio de Janeiro, São Paulo) (Monné 2015a).

***Eburodacrys trilineata* (Aurivillius, 1893)**. Material examined. BRAZIL, *Bahia*: 9 km W Boa Nova (“Caatinga”, 14°36'S, 40°26'W, 750 m), 3 males, 1 female, 4–8.XII.2013, V. O. Becker col. (CVOB).

Described from Brazil (Rio Grande do Sul). According to Martins (1999) the species occurs in Brazil (Minas Gerais, Espírito Santo, São Paulo, Santa Catarina, Rio Grande do Sul).

**ELAPHIDIINI Thomson, 1864**

***Mallocera umbrosa* Gounelle, 1909**. Material examined. BRAZIL, *Bahia*: 9 km W Boa Nova (“Caatinga”, 16°27'S, 39°03'W, 40 m), 2 males, 4–23.XI.2013, V. O. Becker col. (CVOB).

Described from Brazil (Goiás and Minas Gerais). Currently it is known from Brazil (Maranhão, Ceará, Distrito Federal, Goiás, Mato Grosso do Sul, Minas Gerais, São Paulo), Bolivia (Santa Cruz, Tarija), Paraguay, Argentina (Jujuy) (Monné 2015a).

***Stizocera jassuara* (Martins & Napp, 1983)**. Material examined. BRAZIL, *Bahia*: 9 km W Boa Nova (“Caatinga”, 14°36'S, 40°26'W, 750 m), 2 females, 4–8.XII.2013, V. O. Becker col. (CVOB).

Described and it is know from Brazil (Espírito Santo and Rio de Janeiro) (Monné 2015a).

***Stizocera juati* Martins & Napp, 1983**. Material examined. BRAZIL, *Bahia*: 9 km W Boa Nova (“Caatinga”, 14°36'S, 40°26'W, 750 m, 1 male, 1 female, 4–8.XII.2013, V. O. Becker col. (CVOB).

Described from Brazil (Minas Gerais, Espírito Santo, Rio de Janeiro). Currently it is known also from Bolivia (Santa Cruz) (Monné 2015a).

**NEOIBIDIONINI Monné, 2012**

**COMPSINA Martins & Galileo, 2007**

***Engyum vicinum* Martins, Santos-Silva, Galileo & Oliveira 2014**. Material examined. BRAZIL, *Bahia*: 9 km W Boa Nova (“Caatinga”, 14°36'S, 40°26'W, 750 m), male, 4–8.XII.2013, V. O. Becker col. (CVOB).

The species was described and known from Brazil (Maranhão).

**PLEIARTHROCERINI LANE, 1950**

***Pleiarthroceus opacus* Bruch, 1914**. Material examined. BRAZIL, *Bahia*: 9 km W Boa Nova (“Caatinga”, 14°36'S, 40°26'W, 750 m), male, 4–8.XII.2013, V. O. Becker col. (CVOB).

Described from Argentina (Tucumán). Currently it is known from Brazil (Pernambuco, Paraíba, Alagoas), Bolivia (Santa Cruz), Argentina (Salta, Tucumán) (Monné 2015a).

**LAMIINAE Latreille, 1825**

**ACANTHODERINI Thomson, 1860**

***Psapharochrus bivittus* (White, 1855)**. Material examined. BRAZIL, *Bahia*: 9 km W Boa Nova (“Caatinga”, 14°36'S, 40°26'W, 750 m), male, 4–8.XII.2013, V. O. Becker col. (CVOB).

Described from Brazil (Pará). Currently known from Guatemala, Honduras, Nicaragua, Costa Rica, Panama, French Guiana, Brazil (Amazonas, Pará, Maranhão, Espírito Santo to São Paulo), Bolivia (La Paz, Santa Cruz) (Monné 2015b). Zajciw (1968a, 1968b) listed two states in Brazil, not included in Monné (2015b): Pernambuco, and Minas Gerais (a state that it is not clearly placed between Espírito Santo and São Paulo). Marques et al. (2014) recorded that species for the state of Mato Grosso (Brazil).

## Supplementary Material

XML Treatment for Coleoxestia beckeri

XML Treatment for Oncioderes obliqua
